# The genetic susceptibility profile of type 2 diabetes and reflection of its possible role related to reproductive dysfunctions in the southern Indian population of Hyderabad

**DOI:** 10.1186/s12920-021-01129-0

**Published:** 2021-11-16

**Authors:** Kumuda Irgam, Battini Sriteja Reddy, Sai Gayathri Hari, Swathi Banapuram, Battini Mohan Reddy

**Affiliations:** 1 Department of Genetics and Biotechnology, Osmania University, Amberpet, Hyderabad, Telangana 500007 India; 2 Molecular Anthropology Laboratory, Indian Statistical Institute, Street No. 8, Habsiguda, Hyderabad, Telangana 500007 India; 3 Dr Pinnamaneni Siddhartha Institute of Medical Sciences and Research Foundation, Vijayawada, Andhra Pradesh 521286 India

**Keywords:** Complex disorder, Ethnicity, Gender specific, Insulin resistance, Metabolic and reproductive pathways, SNPs

## Abstract

**Background:**

The genetic association studies of type 2 diabetes mellitus (T2DM) hitherto undertaken among the Indian populations are grossly inadequate representation of the ethnic and geographic heterogeneity of the country. In view of this and due to the inconsistent nature of the results of genetic association studies, it would be prudent to undertake large scale studies in different regions of India considering wide spectrum of variants from the relevant pathophysiological pathways. Given the reproductive dysfunctions associated with T2DM, it would be also interesting to explore if some of the reproductive pathway genes are associated with T2DM. The present study is an attempt to examine these aspects in the southern Indian population of Hyderabad.

**Methods:**

A prioritized panel of 92 SNPs from a large number of metabolic and reproductive pathway genes was genotyped on 500 cases and 500 controls, matched for ethnicity, age and BMI, using AGENA MassARRAYiPLEX™ platform.

**Results:**

The allelic association results suggested 14 SNPs to be significantly associated with T2DM at *P* ≤ 0.05 and seven of those—rs2241766-G (ADIPOQ), rs6494730-T (FEM1B), rs1799817-A and rs2059806-T (INSR), rs11745088-C (FST), rs9939609-A and rs9940128-A (FTO)—remained highly significant even after correction for multiple testing. A great majority of the significant SNPs were risk in nature. The ROC analysis of the risk scores of the significant SNPs yielded an area under curve of 0.787, suggesting substantial power of our study to confer these genetic variants as predictors of risk for T2DM.

**Conclusions:**

The associated SNPs of this study are known to be specifically related to insulin signaling, fatty acid metabolism and reproductive pathway genes and possibly suggesting the role of overlapping phenotypic features of insulin resistance, obesity and reproductive dysfunctions inherent in the development of diabetes. Large scale studies involving gender specific approach may be required in order to identify the precise nature of population and gender specific risk profiles for different populations, which might be somewhat distinct.

**Supplementary Information:**

The online version contains supplementary material available at 10.1186/s12920-021-01129-0.

## Introduction

The genetic architecture of T2DM has been widely studied through different approaches and a number of T2DM susceptible genetic variants were identified. While linkage studies traced susceptible variants in genes like CAPN10, TCF7L2, ENPP1, HNF4A, WFS1 and ACDC [1–4] most of which were not subsequently replicated [5]**,** candidate gene studies simultaneously identified PPARG, KCNJ11, TCF7L2, IRS-1, IRS-2, PTPN1, LMNA and few other genes to be consistently associated with T2DM [6]. Further, Genome-wide association studies (GWAS) have identified more than 100 common variants most of which regulate insulin secretion and a few were implicated in insulin sensitivity [6, 7]. Although the understanding of the genetic architecture of T2DM has exhibited considerable progress in the past few years, there may be still many additional genetic variants that might be associated with the pathogenesis of T2DM and further studies are warranted to unveil the underlying pathophysiological mechanisms.

While India is considered as the global diabetic capital, Hyderabad was regarded as the diabetic capital of India. Further, the prevalence of diabetes varies between different states within India and a higher prevalence was observed in the lower economic groups in urban areas of the more economically developed states [8]. However, most of the genetic association studies of T2DM were focused on the North Indian populations [9–11] and with exception to Chennai [12, 13] there were no studies from the Southern regions. Given the high prevalence of T2DM and the unique genetic predisposition of Indians for different complex disorders [14–16], nearly 100 SNPs belonging to 35 genes from different pathways were hitherto screened among the Indian populations, using both candidate gene as well as GWAS approaches. Further, the two GWAS on Indians have identified novel SNPs, rs9552911 (SGCG gene) in Punjabi Sikhs [17] and rs6723108 (TMEM163 gene) in Dravidians and Indo-Europeans [18]. In fact, there were no other genetic studies of type 2 diabetes in the population of Hyderabad when the corresponding author of this manuscript initiated a project in the year 2009 at the Indian Statistical Institute (ISI), Hyderabad, and 15 SNPs belonging to nine genes that were known to be involved in blood-glucose homeostasis—TCF7L2 [19], insulin secretion and action-IGF2BP2 and SLC30A8 [20], insulin signaling pathway and adipocyte differentiation—IRS1, CAPN10 and PPARG [21] and pancreatic beta cell development-CDKAL1, CDKN2A/B and HHEX [22] were genotyped. Further studies of T2DM in Hyderabad were too scanty and focused only on a couple of genes/SNPs [23–25]. Overall, the results among the Indian populations were found to be largely inconsistent with exception to a few SNPs—rs7903146 and rs12255372 of TCF7L2, rs2970847 of PGC-1α, rs9939609 of FTO, rs1801282 of PPARG, rs4402960 of IGF2BP2 and rs5219 of KCNJ11 [9–11, 19, 26–32] that showed a relatively greater degree of consistency in their association with T2DM. In view of the inconsistency observed in the results of genetic association studies of type 2 Diabetes and due to sporadic nature of the studies hitherto conducted in India, it is imperative to explore a large number of SNPs from the relevant genomic regions among different ethnic/geographic populations.

On the other hand, some of the earlier genetic studies have shown that T2DM and polycystic ovary syndrome (PCOS) could share genetic susceptibility factors associated with both pathologies [33] and most PCOS women are vulnerable to develop Type 2 diabetes [34]. However, a number of recent studies among the East Asian [35, 36] and Caucasian [37] populations failed to demonstrate significant association of diabetes genes with PCOS and we tried to test if this could be true for the Indian population as well. We analyzed two different sets of SNPs in the same PCOS cohort from Hyderabad, the first set constituting the same 15 SNPs from the nine diabetes genes earlier genotyped on this T2DM cohort [38] and the second set of additional 92 SNPs of the metabolic and reproductive pathway genes [39]. We observed that neither the diabetes genes nor their interactions with the reproductive pathway genes were significantly associated with PCOS suggesting possible universality of the lack of association [38, 39]. Nevertheless, given that the reproductive dysfunction is a prevalent but less studied complication of T2DM and because of its shared pathophysiology with PCOS, it may be pertinent to examine if the reproductive pathway genes were associated with T2DM and to the best of our knowledge this has not been explored hitherto. In view of the foregoing and given that our earlier research on T2DM in the population of Hyderabad was based on a limited number of 15 SNPs [16], we screened 92 more SNPs, broadly representing both metabolic/diabetes and reproductive pathway genes in the same T2DM cohort in order to portray:

1. The more comprehensive genetic susceptibility profile for Type 2 diabetes in the southern Indian population of Hyderabad;
2. The nature of association of reproductive pathway genes and/or their interactions with diabetic/metabolic pathway genes with T2DM and to explore if that reflects their possible role in the reproductive dysfunction associated with this syndrome.

## Methods

### Study design

The present study is an extension of the research project undertaken by the corresponding author during 2009–2015 at the Indian Statistical Institute, Hyderabad, for which a total sample of 1379 (758 cases and 621controls) [19–22] adult subjects were enrolled after obtaining informed written consent from each of the subjects. The details on the background data, sampling and inclusion and exclusion criteria for enrolling cases and controls were published earlier [21]. Due to resource constraints, we considered random subsets of 500 T2DM cases (290 males + 210 females) and 500 normal controls (350 males + 150 females) of the above set for the present study. The background information of the subjects of the subset is furnished in the Additional file 1: Table S1. The cases and controls were matched for ethnicity, age and BMI. Suffice to mention that the T2DM cases were enrolled from the JP Endocrine Center in Hyderabad and the control subjects were recruited by conducting health camps in different organizations in the same city. The population of Hyderabad is a conglomeration of people from different parts of the undivided state of Andhra Pradesh (AP) and Telugu is one of the four Dravidian languages and spoken by most of the people in AP. Further despite the subdivision of Telugu population into a number of traditionally endogamous castes and sub castes, Reddy et al. [40] observed a very low and insignificant genetic differentiation among the populations of Andhra Pradesh; the Markov chain Monte Carlo analysis of population structure, which implements model-based clustering method for grouping individual into populations [41, 42] did not reveal any unique population clusters, suggesting high degree of genetic homogeneity.

The Indian Statistical Institute Review Committee for Protection of Research Risks to Humans approved the study protocol.

### Isolation of DNA, SNP selection and genotyping

About 3–5 ml of intravenous blood was collected by a trained laboratory technician from each of the T2DM cases and controls. The DNA was isolated using phenol–chloroform method [43] and further quantified with the help of NanoDropTM 2000 (Thermo Scientific, Wilmington, Delaware, USA). Further details on the SNP prioritization, genotyping platform and protocols etc. can be found in one of our recent papers [39]. We may mention that although we attempted genotyping of a prioritized set of 96 SNPs using AGENA MassARRAYiPLEX™ platform at the Imperial Life Sciences Pvt. Ltd., Gurgaon, Haryana, India we could finally consider a panel of only 92 SNPs with call rate > 97% for this study (Additional file 2: Table S2). These 92 SNPs belonged to ~ 40 genes and identified through GWAS and candidate gene studies. While 30 of the 92 SNPs belong to reproductive pathways involved in steroidogenesis and folliculogenesis, the remaining 62 relate to metabolic genes associated with insulin signaling, energy homeostasis and fatty acid metabolism. The primers used for each of the 92 SNPs can be found from our earlier paper [39].

### Statistical analysis

The data pruning, test for Hardy–Weinberg equilibrium (HWE), logistic regression analysis for the allelic association of the individual SNPs, pair-wise SNP–SNP interaction (parametric approach) and haplotype analysis were done with the help of PLINK (version 1.07). After data pruning only 76 of the 92 SNPs that showed minor allele frequency > 1% and conformed to Hardy–Weinberg equilibrium were qualified for further statistical analyses (Additional file 3: Table S3). The *P* value for the association to be significant is set at 0.05 for a single SNP and after Bonferroni correction for multiple testing, it would be p = α/m, where α = 0.05 and m = number of hypotheses or SNPs. It is a very stringent criterion and computes the adjusted *P* values by directly multiplying the number of simultaneously tested hypotheses (*m*), which in this case is 76 SNPs qualified for final analysis. The genotypic association was tested using ‘SNPassoc’ package of R program (version 3.4) and by considering different genetic models—co-dominant, over-dominant, dominant, recessive and log-additive. The model with significant *P* value and lowest AIC (Akaike Information Criterion) was selected as the best fit for the respective SNP. The multiple SNP interaction analysis was done with the help of non-parametric approach by GMDR (version 0.9), where a 10-fold cross-validation with 2, 3, 4 and 5 way interactions were used to detect the gene–gene interactions. Based on the testing balance accuracy and minimal prediction error, the significant interactions were selected. Further, the linkage disequilibrium analysis was performed by Haploview software (version 4.2). The cumulative risk score for each individual was calculated based on the number of significant SNPs and to determine the predictive potential of the risk variants for T2DM, a receiver operating characteristic (ROC) curve was constructed using SPSS (version 25).

## Results

### Allelic and genotypic association

The results of logistic regression analyses suggested 14 of the 76 SNPs to be significantly associated with T2DM at *P* ≤ 0.05 and seven of those remained highly significant even after correction for multiple testing (Table 1) by either Bonferroni single-step adjusted *P* values and/or Benjamini & Hochberg step-up FDR (False Discovery Rate) control. Bonferroni adjustment is one of the most commonly used approaches for multiple comparisons. Six of these seven SNPs involve in insulin signaling, glucose homeostasis, fatty acid metabolism and folliculogenesis. The gene FEM1B is explored for the first time in T2DM and was found to be risk conferring, while it was earlier found to be associated with insulin related traits in PCOS with a protective role [44, 45]. Of the seven highly significant variants, five—rs2241766-G (p_corrected = 2.86 × 10^−34^) of ADIPOQ, rs6494730-T (p_corrected = 6.56 × 10^−13^) of FEM1B, rs2059806-T (p_ corrected = 0.003) of INSR, rs9939609-A (p_ corrected = 0.003) and rs9940128-A (p_ corrected = 0.01) of FTO genes were risk and rs1799817-A (p_corrected = 1.61 × 10^−06^) of INSR and rs11745088-C (p_corrected = 0.001) of FST genes protective in nature. It may be pertinent to note that two of the above seven highly significant SNPs (rs6494730-T and rs11745088-C) belong to reproductive pathway. Interestingly, all the remaining 7 relatively less significant SNPs lost significance after adjusting for covariates—age, sex, migration, family history, physical activity, body mass index and waist hip ratio-, suggesting the confounding nature of the effect of these covariates, particularly the BMI. On the other hand, rs7248104-A of INSR and rs2161829-A of INSIG2 genes that were not significantly associated turned out to be marginally significant (*P* ≤ 0.05) after adjusting for covariates. The results of genotype–phenotype association (Table 2) yielded qualitatively similar results when compared to the allelic association, excepting rs2241766-G, which is an exonic variant and rs6494730-T, an UTR variant, exhibited an increased four-fold and two-fold risk when compared to the degree of their allelic association under the over-dominant model. Although the genotypic association for SNPs was tested under five different models—co-dominant, dominant, over-dominant, recessive and log-additive, the best model with lowest AIC value was considered for the respective SNPs. With exception to one, while all the SNPs showed very good fit under the log additive model, for seven of those it was also the best fitted model for genotypic effects on T2DM. Further, after adjusting for covariates, the significance of most of the SNPs was reduced as observed in the allelic association.

**Table 1:** The results of logistic regression analysis for allelic association of SNP variants with T2DM, unadjusted and adjusted for covariates age, sex, migration, family history, physical activity, BMI, WHR

Gene	SNP rs ID/nature of SNP	Major/minor allele	Minor allele frequency		*χ*^2^	Unadjusted		Adjusted for covariates	
			Cases (N = 500)	Controls (N = 500)		OR (CI 0.95%)	*P* value	OR (CI 0.95%)	*P* value
ADIPOQ	**rs2241766** Non coding trancript variant	T/G	0.41	0.16	158	3.72 (3.01–4.60)	3.14 × 10^–36^	6.62 (4.82–9.08)	1.13 × 10^–31^
FEM1B	**rs6494730** Utr variant	G/T	0.35	0.2	59.18	2.20 (1.80–2.70)	1.44 × 10^–14^	2.34 (1.78–3.08)	1.03 × 10^–09^
INSR	**rs1799817** Exonic	G/A	0.15	0.24	29.05	0.54 (0.43–0.68)	7.07 × 10^–08^	0.53 (0.40–0.70)	1.35 × 10^–05^
	**rs2059806** Intronic	C/T	0.411	0.33	13.12	1.4 (1.17–1.68)	0.0002	1.36 (1.11–1.68)	0.003
	rs7248104 Intronic	G/A	0.43	0.39	3.15	1.17 (0.98–1.40)	0.075^**#**^	1.29 (1.05–1.59)	0.017
FST	**rs11745088** Exonic	G/C	0.01	0.04	15.25	0.28 (0.14–0.55)	9.41 × 10^–05^	0.27 (0.12–0.58)	0.001
FTO	**rs9939609** Intronic	T/A	0.34	0.26	13.67	1.43 (1.18–1.73)	0.0002	1.38 (1.10–1.74)	0.004
	**rs9940128** Intronic	G/A	0.48	0.41	9.374	1.31 (1.10–1.57)	0.002	1.24 (1.00–1.53)	0.042
	rs1421085 Intronic	T/C	0.38	0.33	5.92	1.26 (1.04–1.51)	0.010	1.21 (0.96–1.51)	0.103
	rs17817449 Intronic	T/G	0.35	0.31	4.57	1.22 (1.02–1.48)	0.030	1.22 (0.97–1.53)	0.084
	rs8050136 Intronic	C/A	0.36	0.31	4.55	1.22 (1.02–1.48)	0.030	1.24 (0.99–1.54)	0.065
IRS2	rs12584136 Intronic	C/A	0.03	0.05	5.76	0.55 (0.34–0.90)	0.016	0.63 (0.35–1.12)	0.117
	rs1805097 Missense	C/T	0.03	0.01	5.11	2.03 (1.09–3.80)	0.023	1.54 (0.74–3.18)	0.239
LEPR	rs1805094 Exonic	G/C	0.09	0.12	4.25	0.74 (0.56–0.99)	0.039	0.83 (0.59–1.16)	0.265
c9orf3	rs3802457 Intronic	G/A	0.06	0.09	5.45	0.67 (0.48–0.94)	0.019	0.68 (0.46–1.00)	0.053
INSIG2	rs2161829 Intronic	G/A	0.487	0.45	2.9	1.16 (0.98–1.38)	0.088^**#**^	1.23 (1.00–1.51)	0.048

SNPs in bold are significant after correction for multiple testing

^#^*P* value turns out to be significant after adjusting for covariates

**Table 2:** Genotypic association of SNP variants T2DM, unadjusted and adjusted for age, sex, migration, family history, physical activity, BMI, WHR

SNP	Model	Genotype	Frequency		Unadjusted		Adjusted	
			Cases (500)	Controls (500)	OR (CI 95%)	*P* value	OR (CI 95%)	*P* value
rs2241766	Over dominant	GT	0.81	0.25	12.46 (9.23–16.83)	1.71 × 10^–73^	13.76 (9.39–20.1)	2.47 × 10^–51^
		TT-GG	0.19	0.75	–	–	–	–
rs6494730	Over dominant	GT	0.65	0.31	4.17 (3.2–5.43)	7.94 × 10^–28^	3.73 (2.69–5.16)	5.19 × 10^–16^
		GG-TT	0.35	0.69	–	–	–	–
rs1799817	Log-additive	–	–	–	0.51 (0.4–0.65)	1.63 × 10^–08^	0.53 (0.39–0.71)	2.35 × 10^–05^
rs2059806		–	–	–	1.35 (1.14–1.61)	0.0006	1.4 (1.13–1.74)	0.0019
rs7248104*	Recessive	AA	0.20	0.15	1.38 (0.99–1.92)	0.050	1.28 (1.03–1.59)	0.027
		GG-GA	0.80	0.85	–	–	–	–
rs11745088	Log-additive	–	–	–	0.27 (0.14–0.54)	4.85 × 10^–05^	0.32 (0.14–0.71)	0.003
rs9939609	Dominant	AT-AA	0.58	0.44	1.74 (1.36–2.24)	1.23 × 10^–05^	1.77 (1.29–2.42)	0.0003
		TT	0.42	0.56	–	–	–	–
rs9940128	Log-additive	–	–	–	1.31 (1.10–1.57)	0.002	1.21 (0.97–1.51)	0.089^#^
rs1421085	Dominant	TC-CC	0.64	0.55	1.43 (1.11–1.84)	0.005	1.35 (0.98–1.85)	0.062^#^
		TT	0.36	0.45	–	–	–	–
rs17817449	Log-additive	–	–	–	1.23 (1.02–1.49)	0.029	1.21 (0.96–1.54)	0.109^#^
rs8050136	Log-additive	–	–	–	1.23 (1.02–1.49)	0.030	1.23 (0.97–1.56)	0.080^#^
rs12584136	Over dominant	CA	0.05	0.09	0.52 (0.31–0.87)	0.011	0.54 (0.29–1.03)	0.054^#^
		CC-AA	0.95	0.91	–	–	–	–
rs1805097	Log-additive	–	–	–	2.01 (1.08–3.74)	0.043	1.51 (0.71–3.21)	0.287^#^
rs1805094	Recessive	CC	0.99	0.98	0.20 (0.04–0.9)	0.015	0.28 (0.05–1.60)	0.115^#^
		GG-GC	0.01	0.02	–	–	–	–
rs3802457	Log-additive	–	–	–	0.67 (0.48–0.93)	0.017	0.73 (0.48–1.1)	0.131^#^
rs2161829	Log-additive	–	–	–	1.16 (0.98–1.39)	0.089^#^	1.22 (0.98–1.51)	0.07^#^

^#^*P* value not significant

*Except rs7248104, all other SNPs are significant under log-additive model

In an effort to check the internal consistency of our results and to validate these results in the same population we have split our sample into 30%, 50% and 70% random subsets of cases and controls and repeated the logistic regression analysis of the allelic data. The results suggested similar association pattern with T2DM (Additional file 4: Table S4) in 11 of the 14 significant SNPs in two of the 3 subsets (50% & 70%) as compared to the total sample, thus showing internal consistency and validation of the results. It is not surprising that the 3 SNPs which failed to replicate in the subsets were the ones with only marginally significant association in the total sample.

### SNP–SNP interactions

The logistic regression analysis of the pair wise SNP–SNP interactions through parametric approach yielded a total of 11 highly significant pairs of SNP–SNP interactions (Table 3). Among the 11 pairs of SNP interactions, 4 pairs were significantly associated with risk towards T2DM. Of these, the most significant pair-wise interaction conferring risk towards T2DM was rs1799817-rs2059806 of INSR with an odds ratio of 2.42 and *P* value of 1.91 × 10^−06^. Some of the SNPs which were not significant at the allelic and genotypic associations were found to be significantly associated with T2DM in conjunction with another SNP. It is pertinent to note that out of the total 10 obesity related SNPs from FTO, ADIPOQ and LEPR genes considered in this study, seven were involved in pair-wise interactions and associated with either risk or protective nature towards manifestation of T2DM. The GMDR results for multiple SNP interactions are presented in Table 4. The interaction models above the threshold of testing balance accuracy of 0.55 and with highly significant test *P* values and cross validation consistency were considered to be significant and was observed only at the four loci model involving rs2241766(ADIPOQ), rs2059806 (INSR), rs6494730 (FEM1B) and rs1799817(INSR) SNPs.

**Table 3:** Results of pair wise gene–gene interactions using logistic regression analysis

Gene–Gene	SNP–SNP	OR	*P* value
LEPR-INSR	rs1137101*-rs7248104	1.74	1.89 × 10^–05^
LOC107985940-INSIG2	rs7566605*-rs2161829	1.87	1.13 × 10^–05^
ADIPOQ	rs2241766-rs1501299*	3.13	5.49 × 10^–05^
ADIPOQ-INSR	rs2241766-rs1799817	0.15	1.24 × 10^–12^
DENND1A	rs10818854* rs2479106*	0.20	1.72 × 10^–05^
INSR	rs1799817-rs2059806	2.42	1.91 × 10^–06^
	rs1865434*-rs7987237*	0.37	2.39 × 10^–05^
FTO	rs9940128-rs1421085	0.50	3.92 × 10^–07^
	rs1421085-rs17817449	0.40	9.96 × 10^–11^
	rs1421085-rs8050136	0.40	1.92 × 10^–10^
	rs1421085-rs9939609	0.40	3.77 × 10^–10^

*SNPs only associated at interaction level but not at allelic association

**Table 4:** Results of GMDR analysis involving interactions of multiple SNP combinations

Model	Unadjusted			
	Training balance	Testing balance	Sign test (*P*)	**CVC**
rs2241766	0.77	0.77	10 (0.0010)	10/10
rs2241766 rs5415	0.78	0.77	10 (0.0010)	6/10
rs2241766 rs6165 rs6166	0.79	0.76	10 (0.0010)	5/10
rs2241766 rs2059806 rs6494730 rs1799817	0.81	0.78	10 (0.0010)	10/10
rs2241766 rs2059806 rs6494730 rs1799817 rs2059807	0.84	0.76	10 (0.0010)	3/10

### Linkage disequilibrium (LD) and haplotypes

The pair-wise LD analysis of all the SNPs showed only five SNP pairs with r^2^ > 0.8 and 18 SNP pairs with r^2^ between 0.2 and 0.5. In general, a disrupted pattern of LD was observed with 13 tagged SNPs with r^2^ > 0.8. Using Gabriel haplotype block definition criteria [46], only 7 haplotype blocks could be discerned (Table 5) of which only two were significantly associated with T2DM, one risk conferring after adjusting for covariates (INSR: OR = 1.300, *P* = 0.015) and another protective (FTO: OR = 0.376, *P* = 0.001).

**Table 5:** Haplotype association of variants with T2DM, unadjusted and adjusted for sex, age, migration, physical activity, BMI, WHR, family history

Haplotype block number/ gene	SNPs in the haplotype block	Associated haplotype	Frequency		*χ*^2^	Unadjusted		Adjusted	
			Cases (N = 500)	Controls (N = 500)		OR	*P* value	OR	*P* value
1/PRKAA2	rs11206887 rs2143749	AC	0.19	0.21	0.285	0.941	0.581	0.960	0.783
2/ LEPR	rs1137101 rs1805094	AC	0.09	0.12	3.769	0.755	0.054	0.850	0.345
3/POMC	rs1042571 rs12473543	AT	0.07	0.06	2.187	1.320	0.126	1.320	0.206
3/ IRS2	rs754204 rs7987237	TC	0.45	0.47	0.973	1.070	0.476	0.920	0.444
4/ FTO	rs17817449 rs8050136 rs9939609	GAT	0.02	0.05	14.97	0.376	0.001*	0.410	0.004*
5/INSR	rs2115386 rs1035942	CA	0.31	0.28	0.999	1.100	0.316	1.120	0.302
6/INSR	rs4804416 rs7248104	TA	0.43	0.38	3.167	1.170	0.079	1.300	0.015*
7/FBN3	rs17202517 rs73503752	AT	0.13	0.15	0.424	0.916	0.500	0.910	0.547

*Significant *P* value

### Cumulative risk score analysis

**Fig. 1:**
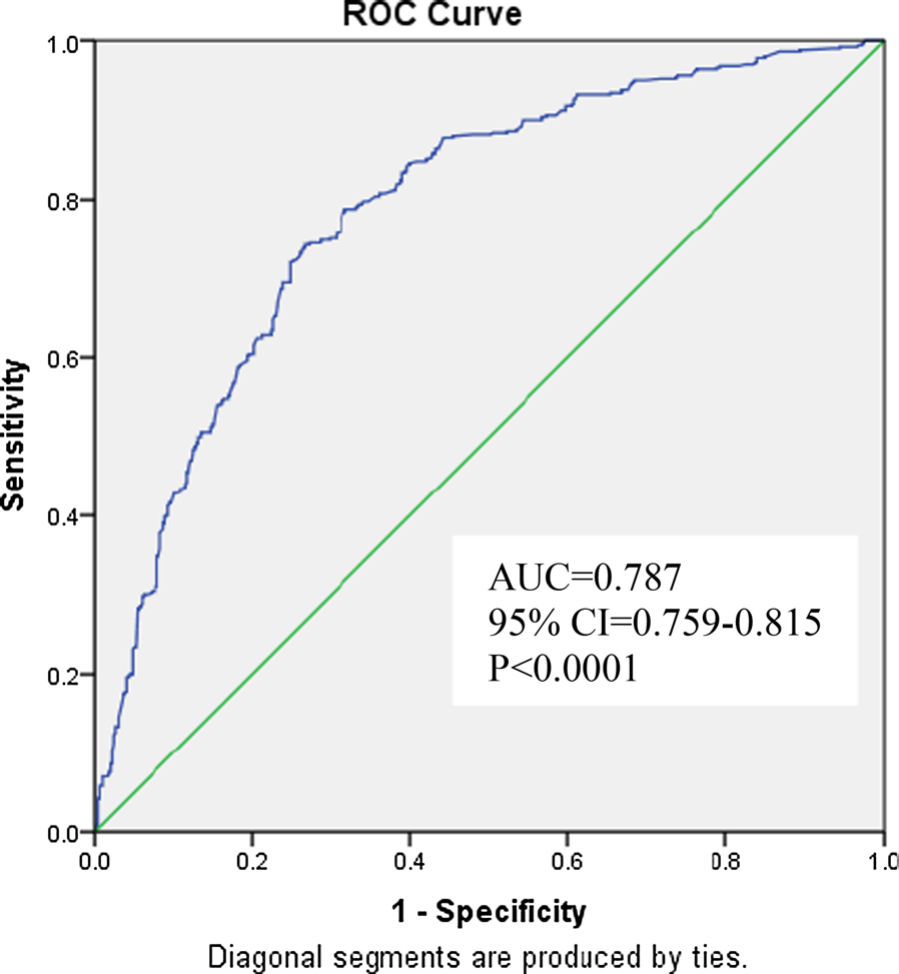
Receiver operating characteristic (ROC) curve indicating the area under curve (AUC) and the discriminative power of risk variants

Based on the 14 significant SNPs, the combined genetic risk score was calculated for each subject by computing the weighted mean proportion of the associated SNPs by considering 2 for two risk alleles, 1 for single risk allele and 0 when there is no risk allele with weights as relative log odds ratios with respect to different SNPs. The cumulative risk score for each individual was obtained by multiplying the combined genetic risk score with the number of significant SNPs. The individuals with risk scores ranging from 8.0 to 21.9 were grouped into 10 risk categories and the number of cases and controls in each category are presented in Table 6, which suggests a translucent trend of increased proportion of cases when compared to the controls with increasing risk score/category, particularly from the 6th. This has also reflected in the increasing trend of odds ratios computed by using risk category 1 as the reference. The odds ratios for the risk categories 4–10 were found to be significantly associated with T2DM. The ROC curve was constructed and the area under curve (AUC) was observed to be highly significant [0.787 (95% CI 0.759–0.815, *P* ≤ 0.0001)] (Fig. 1), which may suggest the predictive utility of these risk variants for T2DM in this population. Further, the ROC curve based on the risk score of seven highly significant SNPs (after correction for multiple testing) and/or for only five of the 7 with risk yielded very similar AUC and the predictive value as to that of 14 SNPs.

**Table 6:** Risk score analysis according to the cumulative risk score for T2DM cases and controls

Risk category (Risk score)	% of individuals in Cases (N = 500)	% of individuals in Controls (N = 500)	Odds ratio (95% CI)	Z score	*P* value
1 (8–10.9)	1.40	11.4	Reference	–	–
2 (11–11.9)	2.40	12.6	1.55 (0.57–4.21)	0.407	0.389
3 (12–12.9)	3.60	15.0	1.95 (0.76–4.99)	0.907	0.162
4 (13–13.9)	4.40	12.2	2.99 (1.18–7.52)	1.089	0.020
5 (14–14.9)	11.0	17.6	5.03 (2.14–11.8)	1.713	0.001
6 (15–15.9)	16.8	11.0	12.4 (5.28–29.2)	2.544	0.001
7 (16–16.9)	17.4	9.20	15.0 (6.34–35.6)	2.702	0.001
8 (17–17.9)	20.8	6.20	27.8 (11.5–67.2)	3.195	0.001
9 (18–18.9)	10.6	2.40	35.9 (13.2–98.0)	3.164	0.001
10 (19–21.9)	11.6	2.40	39.3 (14.5–107)	3.238	0.001

### Genetic association patterns in the male and female cohorts

The heterogeneity in biology, environment and lifestyle pattern determine the distinction in the genetic predisposition and development of any complex disease among males and females [47]. Likewise, the disparities among males and females in sex hormones have an important effect on T2DM as observed in case of androgens, since hyperandrogenism in females and hypogonadism in males are prominent risk factors for T2DM [48]. Further, given the shared pathophysiology of T2DM and PCOS and since majority of PCOS women manifest T2DM at later stage and even a notable fraction of younger T2DM female show signs of PCOS [33] one would surmise if the genetic variants associated with reproductive phenotype of PCOS would also be associated with T2DM patients. The results of association analyses in gender specific cohorts (Table 7) suggested significant association of 14 and 13 SNPs, respectively in males and females. Further, while eight of the 14 SNPs were significant even after correction for multiple testing in males only two SNPs were significant in the female subset, which could be partly due to relatively small sample size for females. Further, seven SNPs of the male subset and nine SNPs of the female subset were found to be associated with risk towards T2DM. While 11 of the 14 significant SNPs from the male subset are common with that of the pooled set (Males + females), only seven of the 13 SNPs from the female subset were common, reflecting that 78% of the pooled set was represented by males and hence the pattern in males is more complementary to the pooled set. Interestingly, the SNPs of PRKAG3, ESR2 and FBN3 genes not associated in the pooled set, were observed to be significantly associated in the male and/or female subsets; while the SNP of PRKAG3 was protective in females, the variants of reproductive pathway genes—FBN3 showed protective nature in males and risk in females and ESR2 was protective in both the sub sets.

**Table 7:** Summary of allelic association results in the male and female subsets of T2DM

S. No	Gene	SNP	T2DM males*		T2DM females*	
			Odds ratio	*P* value	Odds ratio	*P* value
**Diabetic genes**						
1	INSR	rs1799817	0.49(0.37–0.66)	**1.85 × 10**^**–06**^	0.61(0.42–0.89)	0.010
		rs2059806	1.38(1.10–1.74)	**0.004**	1.45(1.06–1.98)	0.018
		rs2115386^ǂ^	–	–	1.50(1.11–2.02)	0.007
		rs1035942^ǂ^	–	–	1.48(1.07–2.06)	0.017
2	IRS2	rs12584136	–	–	0.38(0.16–0.89)	0.021
		rs1805097	2.04(0.99–4.22)	0.047	–	–
3	INSIG2	rs2161829^ǂ^	–	–	1.41(1.05–1.90)	0.021
**Obesity genes**						
4	ADIPOQ	rs2241766	4.00(3.06–5.22)	**5.42 × 10**^**–26**^	3.14(2.21–4.45)	**4.77 × 10**^**–11**^
5	FTO	rs9939609	1.51(1.19–1.91)	**0.001**	–	–
		rs9940128	1.29(1.04–1.61)	0.021	1.46(1.08–1.99)	0.013
		rs1421085	1.31(1.05–1.65)	0.017	–	–
		rs17817449^**#**^	–	–	–	–
		rs8050136^**#**^	–	–	–	–
6	LEPR	rs1805094	0.68(0.47–0.98)	0.041	–	–
**Reproductive genes**						
7	FEM1	rs6494730	2.40(1.85–3.12)	**2.34 × 10**^**–11**^	1.76(1.27–2.45)	**0.001**
8	FST	rs11745088	0.14(0.05–0.42)	**4.44 × 10**^**–05**^	–	–
9	FBN3	rs17202517^ǂ^	0.67(0.49–0.91)	0.010	1.73(1.13–2.67)	0.011
		rs73503752^ǂ^	0.72(0.53–0.99)	0.048	1.59(1.02–2.46)	0.036
10	ESR2	rs1256049^ǂ^	0.58(0.41–0.82)	**0.002**	0.51(0.32–0.80)	0.003
**Other genes**						
11	PRKAG3	rs6436094^ǂ^	–	–	0.68(0.50–0.94)	0.021
12	c9orf3	rs3802457	0.55(0.36–0.83)	**0.004**	–	–

*P* values in bold indicate significant after correction for multiple testing

^ǂ^Found only in subsets

^#^Associated only in pooled set

*Sample size: Male cohort—290 cases and 350 controls; female cohort—210 cases and 150 controls

## Discussion

Fourteen of the 92 SNPs were observed to be significantly associated with T2DM (*P* ≤ 0.05) in the present cohort of southern Indians from Hyderabad of which nine were risk conferring and five showed protective nature. While seven of the 14 significant SNPs belong to obesity genes, four from insulin signaling pathway, two from reproductive pathway genes and one plays a role in peptide hormone metabolism. The pathophysiological link between obesity and T2DM primarily relates to the adipose tissue that regulates appetite and metabolism [49]. Adiponectin is expressed in adipose tissue and low levels of it may cause reduction of fatty acid oxidation, elevated plasma glucose and insulin resistance, ultimately leading to T2DM [50]. The rs2241766-G of ADIPOQ gene significantly associated in this study was found to be associated with T2DM in another sample of south Indians [51] and in different other ethnic groups outside India—in Japanese [52], Singaporean Chinese [53], Han Chinese [54] and Iraqi populations [55]. Furthermore, the in-silico analysis also revealed that + 45 T>G polymorphism causes a synonymous change (G15G) and modulates the expression of adiponectin gene via affecting the splicing machinery [56]. Another obesity gene leptin, a precursor of adipokinases, is also secreted by adipose tissue. Leptin activates its receptor (LEPR) in the hypothalamus to alter the expression of several neuropeptides which regulate appetite and metabolism. Also, the presence of LEPR in pancreatic β-cells may be involved in the onset of chronic hyperglycemia and uncontrolled T2DM [57]. But the rs1805094-C of this gene was associated with protective nature towards T2DM in this study. Additionally, the FTO variants associated in this study include—rs9939609-A, rs9940128-A, rs1421085-C, rs17817449-G and rs8050136-A. Although the functional role of FTO gene in predisposing T2DM is unclear, it is presumed to code for 2-oxo-glutarate-dependent demethylase enzyme [58] which influences nucleic acid demethylation and may be important in epigenetic regulation. Further, earlier studies among the northern [10] and western Indian populations [11] demonstrated that the SNPs of FTO gene predispose South Asian Indians to T2DM and unlike in Europeans they do not appear to do this entirely through their influence on BMI; even after adjusting for BMI, these SNPs showed significant association with T2DM [59] and this has been the case with the present study as well. While the rs9939609 showed association in the North and Western Indian populations [10, 11] as well as in Palestinians [60], rs9940128-A and rs8050136-A were found to be associated with T2DM in the population of Chennai, [61] and rs1421085-C not associated in the North Indian sample [62]. The 3 variants-rs1421085, rs17817449 and rs8050136-that lost significance after adjusting for BMI, suggest that unlike in the case of rs9939609 and rs9940128 their association with T2DM possibly linked through obesity**.**

The IRS-2 located on chromosome 13q34 is primarily a progesterone response gene, mediates glucose metabolism and tumor progression [63]. The rs1805097-T polymorphism of IRS2 was associated with risk in this study. It was predicted that this non-synonymous change (GLY1057ASP) might affect transcriptional regulation, splicing and post transcriptional modifications. However, the other SNP rs12584136-A of IRS-2 was associated with protective nature towards T2DM. Likewise, the INSR gene encodes the insulin receptor which acts as mediator between the extracellular and intracellular insulin signaling pathways and is essential for the action of insulin. Further, INSR is involved in adipogenesis and beta-cell insulin secretion and mutations in this gene leads to T2DM [64]. Concurrently, the rs2059806 of INSR gene which is located in the coding region of exon 8 and known to cause synonymous change was found to be associated with risk for T2DM in the present study and in Han-Chinese [65]. Whereas another SNP of INSR, rs1799817-A, showed protective nature of association towards T2DM in our study and in another sample of south Indians from Chennai [66], it was not associated in Han-Chinese population [65].

The etiology of T2DM and PCOS share many common aspects, for instance genetic predisposition, insulin resistance and obesity [67]. In this context, it was thought pertinent to test the association of some of the reproductive genes that are putatively responsible for PCOS with T2DM. We observed four reproductive genes to be associated in the subsets, of which rs6494730-T, a 3’utr variant of FEM1B gene associated with risk towards T2DM in the pooled as well as male and female subsets suggesting highly significant risk for both the sexes. This gene is a homologue of nematode sex determination gene, expressed in skeletal muscles and pancreas which regulates plasma glucose levels and insulin secretion [68]. Further, the gender specific analysis also suggested association of reproductive pathway gene variants, other than those associated in the pooled cohort -FBN3 and ESR2. The FBN3 gene which is associated with significant risk for T2DM females in the present study was also found to be highly expressed in fetal tissues and retaining its low levels in post natal tissues [69]. Further, this gene was also known to be associated with secondary amenorrhea in PCOS [70], hence its presumptive role in reproductive abnormalities of T2DM females as well. On the other hand, the gender specific association patterns are found to be not that distinct in the sense most of the associated genes are common to both male and female cohorts albeit certain SNPs within genes were observed to be significantly associated in either males or females, not in both. A couple of genes whose association was restricted to one of the two sexes happened to be protective in nature (Table 7). However, only specifically designed large scale gender specific studies can provide clear cut picture on the possibility of gender specific susceptibility profiles.

**Fig. 2:**
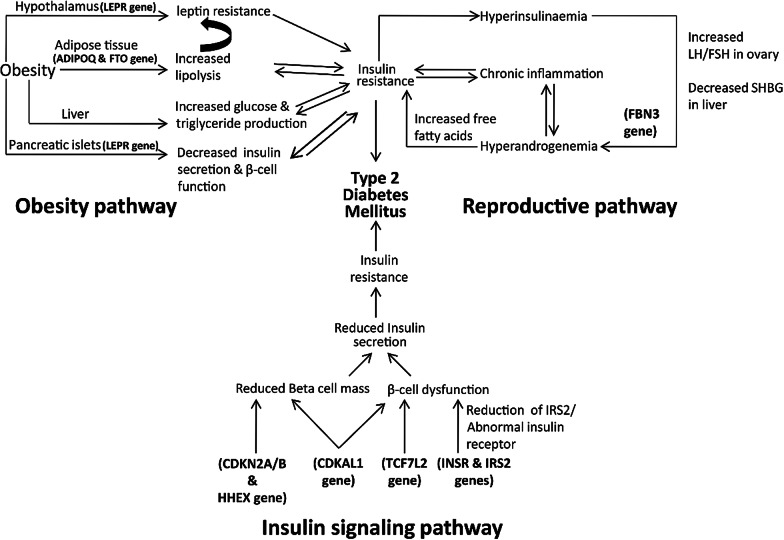
Schematic diagram illustrating the putative role of different molecular mechanisms involved in the major pathophysiological pathways of type 2 diabetes

Overall, the results of the present study based on 92 SNPs suggest that the SNPs associated with T2DM mostly belong to the genes related to obesity and insulin signaling pathways and supplemented with the results of our earlier study of 15 SNPs from the 9 most important T2DM genes on the same cohort (Additional file 5: Table S5) provide by far the most comprehensive susceptibility profile for any major regional population/ethnic group hitherto studied in India with a possible exception to a very few [30] and the two recent GWAS each focused on a regional ethnic/linguistic group [17, 18]. Although our earlier study was based on much larger cohort (758 cases and 621 controls as against the random subset of 500 cases and 500 controls for the present study of 92 SNPs), we reanalyzed the 15 SNP data in the reduced random subset and obtained qualitatively and quantitatively very similar results, in that seven of the 15 SNPs were found to be significant (*P* ≤ 0.05), risk conferring to T2DM and belong to TCF7L2, CDKAL1, IRS1 and CAPN10 genes. Two of the 3 SNPs of TCF7L2 and the two SNPs of CDKAL1 genes were found to be significant even after correction for multiple testing. However, none of the pair wise interactions of the 15 SNPs with that of the 92 of the present study were found to be significantly associated with the disease albeit only one 5-loci interaction model, containing rs2059806, rs2241766, rs6494730, rs1799817 and rs7903146, was found to be significant in which only rs7903146 of TCF7L2 from the 15 SNP set was a constituent. To sum up, the overall genetic susceptibility profile of T2DM in the population of Hyderabad broadly represents the genes that might possibly interrupt homeostasis including insulin action and sensitivity, β-cell function and their proliferation and fatty acid oxidation as illustrated in the schematic diagram (Fig. 2). Although, the precise nature of the role of reproductive genes associated in this study cannot be readily discerned, they possibly manifest T2DM through hyperandrogenism and defects in insulin secretion. It can be also presumed that the risk of developing T2DM in PCOS women is identical to that of the manifestation of PCOS features in T2DM women at the reproductive stage. The functional validation of some of these most significant SNPs should have definitely added strength to the inference on the role of risk variants in the development of T2DM, excepting for the fact that most of them are intronic variants and two of the three exonic variants observed in this study happened to be protective in nature. The only utr variant (rs6494730) associated was studied for the first time with reference to T2DM. It would be prudent to pursue functional validation of this SNP in near future.

## Conclusions

Of the 14 significantly associated SNPs, rs2241766-G of ADIPOQ was the most prominent one with risk towards T2DM and was also present at all levels of interactions. On the other hand, the lack of association of certain SNPs in this cohort need not necessarily rule out the possibility of their role in other ethnic groups given that the frequency of the susceptible genetic variants may vary from population to population. There are indications from our study that the gender specific susceptibility profiles might be possible albeit specifically designed large scale studies are required to confirm this observation. Given this, the analyses of the pooled cohorts of male and female samples may cause confounding effects leading to the distorted picture of susceptibility profile of a particular ethnic/regional population and unfortunately this has been the pattern of association studies hitherto, be it diabetes or any other complex disease. The association of certain reproductive and insulin/obesity related SNPs in this study reflects the shared etiologies of T2DM and PCOS. Given this, exploring this angle in the susceptibility profile of T2DM with the help of specifically designed large scale gender specific studies in different ethnic and geographic populations of the country would not only help unraveling the role of reproductive genes in predisposing T2DM but also provide gender specific susceptibility profiles for this disease if existent.

## Supplementary Information

**Additional file 1: Table S1.** Comparison of baseline characteristics of T2DM cases and controls

**Additional file 2: Table S2.** List of genes with SNPs included in this study along with characteristic feature, position and the putative function

**Additional file 3: Table S3.** Allelic association results of the 92 SNPs considered in this study

**Additional file 4: Table S4.** Allelic association results of 70% and 50% random subsets in comparison to the total cohort

**Additional file 5: Table S5.** The results of logistic regression analyses of the 15 SNP earlier studied in the same cohort and the patterns of association with T2DM, unadjusted and adjusted for covariates

## Data Availability

The data can be made available on reasonable request to the corresponding author. Most of the data are furnished in the form of additional files tables.

## Abbreviations

T2DM: Type 2 diabetes mellitus
GWAS: Genome wide association studies
PCOS: Polycystic ovary syndrome
ROC: Receiver operating characteristic
AUC: Area under curve
