# Supplementary material for: The genetic susceptibility profile of type 2 diabetes and reflection of its possible role related to reproductive dysfunctions in the southern Indian population of Hyderabad
Source: BMC Med Genomics. 2021 Nov 16;14:272. doi: 10.1186/s12920-021-01129-0 (PMC8597259; doi:10.1186/s12920-021-01129-0)
Supplement: Supplementary file 2 — Additional file 2: Table S2. List of genes with SNPs included in this study along with characteristic feature, position and the putative function [file 12920_2021_1129_MOESM2_ESM.docx]

**Additional file 2 Table S2: List of genes with SNPs included in this study along with characteristic feature, position and the putative function**

| **Gene** | **Locus** | **SNP** | **Major/**  **Minor allele** | **SNP position** | **Characteristic feature** | **Function** |
| --- | --- | --- | --- | --- | --- | --- |
| **Reproductive pathways**  LHCGR  (Luteinizing hormone/choriogonadotropin receptor) | 2p16.3 | rs13405728 | A/G | 48751020 | Intron variant | Development of Leydig cells |
| FBN3  (FIBRILLIN 3) | 19p13.2 | rs17202517 | G/A | 8083417 | Intron variant | Integrin Pathway and ERK Signalling |
|  |  | rs73503752 | C/T | 8084117 | Intron variant, nc transcript variant, Synonymous codon |  |
| FEM1A  (FEM-1 homolog A) | 13q14.3 | rs111933 | C/T | 50759929 | Intron variant | Controlsmasculinization |
| FEM1B  (FEM-1 homolog B) | 15q23 | rs6494730 | G/T | 68292218 | utr variant 3 prime | Mediates Apoptosis |
| FSHR  (Follicle stimulating hormone receptor) | 2p16.3 | rs6165 | C/T | 48963902 | Missense | Gonad development |
|  |  | rs6166 | T/C | 48962782 | Missense |  |
|  |  | rs2268361 | T/C | 48974473 | Intron variant |  |
| FST  (Follistatin) | 5q11.2 | rs1127760 | 0 | 53484287 | Missense | TGF-beta Receptor Signaling and DNA Damage/Telomere Stress Induced Senescence |
|  |  | rs722910 | T/A | 53485767 | Intron variant |  |
|  |  | rs3797297 | G/A | 53481826 | Intron variant |  |
|  |  | rs11745088 | 0 | 53483680 | Missense |  |
| ADRB2  (Adrenoceptor beta 2) | 5q32 | rs1042714   \|  \| \| --- \| \|  \| | C/G | 148826910 | Missense | Metabolism of proteins and monoamine GPCRs |
|  |  | rs1042713 | G/A | 148826877 | Missense |  |
| ACVR2A  (Activin A receptor type 2A) | 2q22.3-q23.1 | rs1424941 | C/T | 147885549 | Intron variant | Mediates the functions of  Activins |
|  |  | rs3768688 | A/G | 147913532 | Intronvariant |  |
| ESR1  (Estrogen Receptor 1 ) | 6q25.1-q25.2 | rs1514348 | T/G | 151861180 | Intron variant | Estrogen receptor pathway |
|  |  | rs3020314 | C/T | 151949537 | Intron variant |  |
| ESR2  (Estrogen Receptor 2) | 14q23.2-q23.3 | rs1256049 | C/T | 64257333 | Intron variant | Estrogen receptor pathway |
| HSD17B6  (Hydroxysteroid 17-beta dehydrogenase 6) | 12q13.3 | rs898611 | T/C | 56769532 | Intron variant | Androgen Catabolism |
| HSD17B5/ AKR1C3  (Aldo-ketoreductase family 1 member C3) | 10p15.1 | rs1937845 | G/A | 5093956 | Intron variant, upstream variant 2KB | AKR1C3 generates testosterone and progesterone, catalyzesthe conversion of aldehydes and ketones into alcohols. |
|  |  | rs12529 | G/C | 5094459 | Intron variant, missense |  |
| PGR  (Progesterone receptor) | 11q22.1 | rs1042838 | C/A | 101062681 | Intron variant, Missense, nc transcript variant | Mediates the physiological effects of Progesterone. |
|  |  | rs10895068 | C/T | 101129483 | nc transcript variant, Upstream variant 2KB, Utr variant 5 prime |  |
| POMC  (Proopiomelanocortin) | 2p23.3 | rs1042571 | G/A | 25161018 | Utr variant 3 prime | Peptide hormone metabolism and Aldosterone synthesis and secretion |
|  |  | rs12473543 | T/G | 25164312 | Intron variant |  |
| SHBG  (Sex hormone binding globulin) | 17p13.1 | rs1799941 | G/A | 7630105 | Intron variant  Utr variant 5 prime | Regulation of steroids |
|  |  | rs6258 | C/T | 7631360 | Missense |  |
|  |  | rs6259 | G/A | 7633209 | Missense, Synonymous codon |  |
| **Metabolic**  **pathways**  INSR  (Insulin Receptor) | 19p13.2 | rs7248104 | G/A | 7224420 | Intron variant | Insulin receptor signaling and Glucose homeostasis |
|  |  | rs1035942 | G/A | 7199792 | Intron variant |  |
|  |  | rs12971499 | T/C | 7214271 | Intron variant |  |
|  |  | rs2059806 | C/T | 7166365 | Exonic |  |
|  |  | rs2115386 | C/T | 7196554 | Intron variant |  |
|  |  | rs2252673 | G/C | 7150407 | Intron variant |  |
|  |  | rs891088 | A/G | 7184751 | Intron variant |  |
|  |  | rs10401628 | G/A | 7126207 | Intron variant |  |
|  |  | rs12459488 | C/G | 7206062 | Intron variant |  |
|  |  | rs1799817 | G/A | 7125286 | Exonic |  |
|  |  | rs2059807 | G/A | 7166098 | Intron variant |  |
|  |  | rs4804416 | T/G | 7223837 | Downstream variant 500B,intron variant |  |
| ADIPOQ  (Adiponectin, C1Q and Collagen Domain Containing) | 3q27.3 | rs2241766 | T/G | 186853103 | Nc transcript variant, synonymous codon | Adiponectin signaling |
|  |  | rs1501299 | G/T | 186853334 | Intron variant |  |
| LEPR  (Leptin receptor) | 1p31.3 | rs1137100 | A/G | 65570758 | Missense | Energy homeostasis and obesity |
|  |  | rs1137101 | G/A | 65592830 | Benign |  |
|  |  | rs1805094 | G/C | 65610269 | Missense |  |
| PRKAA2  (protein kinase AMP-activated catalytic subunit alpha 2) | 1p32.2 | rs12749128 | 0 | 56688704 | Intron variant | Cellular energy metabolism |
|  |  | rs2746349 | T/C | 56660977 | Intron variant |  |
|  |  | rs11206887 | G/A | 56651236 | Intron variant |  |
|  |  | rs2143749 | C/G | 56656794 | Intron variant |  |
|  |  | rs2051040 | C/T | 56677082 | Intron variant |  |
|  |  | rs3738568 | A/G | 56707918 | nc variant |  |
| PRKAG3  (protein kinase AMP-activated non-catalytic subunit gamma 3) | 2q35 | rs16859382 | T/C | 218829133 | Intron variant | Cellular energy metabolism |
|  |  | rs6436094 | A/G | 218822874 | Upstream variant |  |
|  |  | rs650898 | A/G | 218831291 | Intron variant |  |
| AKT2  (AKT serine/threonine kinase 2) | 19q13.2 | rs11671439 | T/C | 40272270 | Intron variant | Metabolism, proliferation, cell survival, growth and angiogenesis. |
|  |  | rs2304188 | T/C | 40236198 | Intron variant |  |
|  |  | rs3730051 | T/C | 40238790 | Intron variant |  |
|  |  | rs8100018 | G/C | 40246116 | Intron variant |  |
| IRS2  (Insulin receptor substrate 2) | 13q34 | rs12584136 | C/A | 109767007 | Intron variant | Insulin signaling |
|  |  | rs2289046 | T/C | 109755559 | Utr variant 3 prime |  |
|  |  | rs754204 | C/T | 109759221 | Intron variant |  |
|  |  | rs7997595 | C/G | 109778421 | Intron variant |  |
|  |  | rs137852740 | 0 | 109784115 | Missense, Synonymous codon |  |
|  |  | rs1865434 | T/C | 109756261 | Utr variant 3 prime |  |
|  |  | rs1805097 | C/T | 109782884 | Missense |  |
|  |  | rs7987237 | C/T | 109762666 | Intron variant |  |
| FTO  (FTO, alpha-ketoglutarate dependent dioxygenase) | 16q12.2 | rs17817449 | T/G | 53779455 | Intron variant | Adipogenesis and obesity associated. |
|  |  | rs8050136 | C/A | 53782363 | Intron variant |  |
|  |  | rs1421085 | T/C | 53767042 | Intron variant |  |
|  |  | rs9930506 | AG | 53796553 | Intron variant |  |
|  |  | rs9939609 | T/A | 53786615 | Intron variant |  |
|  |  | rs9940128 | G/A | 53766842 | Intron variant |  |
| INSIG2  (Insulin induced gene 2) | 2q14.1-q14.2 | rs2161829 | G/A | 118099338 | Intron variant | Regulation of cholesterol biosynthesis by SREBP (SREBF) and Metabolism |
| MTCH2  (Mitochondrial Carrier  Homolog 2) | 11p11.2 | rs10838738 | A/G | 47641497 | Intron variant | Adipocyte differentiation |
| SH2B1  (SH2B adaptor protein 1) | 16p11.2 | rs7498665 | A/G | 28871920 | Intron variant, Missense, nctranscript variant | Cytokine and  growth factor receptor signaling  and cellular transformation |
| THADA  (Thyroid adenoma associated ) | 2p21 | rs12478601 | T/C | 43494369 | Intron variant | Involved in lipid homeostasis and regulates organismal balance between energy storage and heat production. |
|  |  | rs13429458 | A/C | 43411699 | Intron variant |  |
| DENND1A  (Differentially expressed in Normal and Neoplastic Development isoform 1A) | 9q33.3 | rs2479106 | A/G | 123762933 | Intron variant | Functions as a guanine nucleotide exchange factor that interacts with RAB family and involves in clatharin mediated endocytosis. |
|  |  | rs10818854 | G/A | 123684499 | Intron variant |  |
| c9orf3  (Chromosome 9 Open Reading Frame 3) | 9q22.32 | rs3802457 | G/A | 94979054 | Intron variant | Metabolism of peptide hormones and proteins. |
| KHDRBS3  (KH domain containing, RNA binding, signal transduction-associated 3 gene | chromosome no 8 | rs10505648 | A/G | 136144207 | Upstream | Telomerase activity and Regulates alternate splicing |
| YAP1  (Yes associated protein 1) | 11q22.1 | rs1894116 | A/G | 102199908 | Intron variant | Hippo signalling pathway |
| SLC2A4  (Solute carrier family 2 member 4) | 17p13.1 | rs5415 | C/T | 7281162 | Upstream variant 2KB | Insulin-regulated facilitative glucose transporter |
|  |  | rs5417 | C/A | 7281743 | Utr variant 5 prime |  |
|  |  | rs5435 | T/C | 7283804 | Missense, synonymous codon |  |
| LOC107984901  (Uncharacterized LOC107984901)  Near TOX3 | 16q12.1 | rs4784165 | T/G | 52313907 | Intergenic | -- |
| RAB5B,SUOX  (RAB5B, member RAS oncogene family, Sulphite Oxidase) | Chromosome  no. 12 | rs705702 | A/G | 55996852 | Upstream variant | Metabolism of proteins and involves in Innate immune system |
| SOX8 | chromosome no.16 | rs500492 | T/C | 1000877 | Exonic | May involve in the development of brain |
| LOC107985940  (Uncharacterized LOC107985940) | 2q14.1 | rs7566605 | G/C | 118078449 | Intron variant | -- |
| LINC01060  (long intergenic non-protein coding RNA 1060)  (Near TRIML1) | 4q35.2 | rs7666129 | T/C | 188576910 | Intron variant | -- |
| SUMO1P1  (SUMO1 pseudogene 1) | 20q13.2 | rs6022786 | G/A | 53830764 | Regulatory variant | -- |
