# Supplementary material for: The genetic susceptibility profile of type 2 diabetes and reflection of its possible role related to reproductive dysfunctions in the southern Indian population of Hyderabad
Source: BMC Med Genomics. 2021 Nov 16;14:272. doi: 10.1186/s12920-021-01129-0 (PMC8597259; doi:10.1186/s12920-021-01129-0)
Supplement: Supplementary file 4 — Additional file 4: Table S4. Allelic association results of 70% and 50% random subsets in comparison to the total cohort [file 12920_2021_1129_MOESM4_ESM.docx]

**Additional file 4 Table S4: Allelic association results of 70% and 50% random subsets in comparison to the total cohort**

| **S.No** | **Gene** | SNP | **%Sample** | **Allele** | **MAF** | | **Odds ratio (95%C.I)** | **p value** |
| --- | --- | --- | --- | --- | --- | --- | --- | --- |
|  |  |  |  |  | **Cases** | **Controls** |  |  |
|  |  |  |  |  |  |  |  |  |
| 1 | ADIPOQ | rs2241766 | Total | T/G | 0.41 | 0.16 | 3.72(3.01-4.60) | 3.14x10^-36^ |
|  |  |  | 70 |  | 0.42 | 0.15 | 4.01(3.12-5.16) | 6.69x10^-29^ |
|  |  |  | 50 |  | 0.42 | 0.16 | 3.92(2.90-5.28) | 2.48x10^-20^ |
| 2 | FEM1B | rs6494730 | Total | G/T | 0.35 | 0.20 | 2.20(1.80-2.70) | 1.44x10^-14^ |
|  |  |  | 70 |  | 0.34 | 0.18 | 2.34(1.83-2.99) | 6.46x10^-12^ |
|  |  |  | 50 |  | 0.36 | 0.21 | 2.13(1.61-2.82) | 9.89x10^-08^ |
| 3 | INSR | rs1799817 | Total | G/A | 0.15 | 0.24 | 0.54(0.43-0.68) | 7.07x10^-08^ |
|  |  |  | 70 |  | 0.14 | 0.24 | 0.49(0.37-0.64) | 2.55x10^-07^ |
|  |  |  | 50 |  | 0.15 | 0.26 | 0.49(0.36-0.67) | 9.71x10^-06^ |
| 4 | INSR | rs2059806 | Total | C/T | 0.41 | 0.33 | 1.4(1.17-1.68) | 0.0002 |
|  |  |  | 70 |  | 0.40 | 0.32 | 1.43(1.15-1.77) | 0.001 |
|  |  |  | 50 |  | 0.40 | 0.32 | 1.40(1.08-1.82) | 0.01 |
| 5 | FST | rs11745088 | Total | G/C | 0.01 | 0.04 | 0.28(0.14-0.55) | 9.41x10^-05^ |
|  |  |  | 70 |  | 0.01 | 0.04 | 0.22(0.09-0.53) | 0.0003 |
|  |  |  | 50 |  | 0.01 | 0.03 | 0.26(0.08-0.80) | 0.012 |
| 6 | FTO | rs9939609 | Total | T/A | 0.34 | 0.26 | 1.43(1.18-1.73) | 0.0002 |
|  |  |  | 70 |  | 0.34 | 0.25 | 1.53(1.21-1.92) | 0.0002 |
|  |  |  | 50 |  | 0.35 | 0.26 | 1.57(1.20-2.06) | 0.001 |
| 7 | FTO | rs9940128 | Total | G/A | 0.48 | 0.41 | 1.31(1.10-1.57) | 0.002 |
|  |  |  | 70 |  | 0.47 | 0.39 | 1.39(1.12-1.71) | 0.002 |
|  |  |  | 50 |  | 0.50 | 0.40 | 1.55(1.20-1.98) | 0.001 |
| 8 | FTO | rs1421085 | Total | T/C | 0.38 | 0.33 | 1.26(1.04-1.51) | 0.014 |
|  |  |  | 70 |  | 0.37 | 0.31 | 1.33(1.06-1.65) | 0.012 |
|  |  |  | 50 |  | 0.42 | 0.31 | 1.57(1.21-2.03) | 0.001 |
| 9 | FTO | rs17817449 | Total | T/G | 0.35 | 0.31 | 1.22(1.02-1.48) | 0.032 |
|  |  |  | 70 |  | 0.35 | 0.28 | 1.34(1.07-1.68) | 0.009 |
|  |  |  | 50 |  | 0.36 | 0.30 | 1.31(1.00-1.71) | 0.046 |
| 10 | FTO | rs8050136 | Total | C/A | 0.36 | 0.31 | 1.22(1.02-1.48) | 0.033 |
|  |  |  | 70 |  | 0.35 | 0.29 | 1.33(1.06-1.66) | 0.013 |
|  |  |  | 50 |  | 0.36 | 0.30 | 1.31(1.00-1.70) | 0.046 |
| 11 | IRS2 | rs12584136 | Total | C/A | 0.03 | 0.05 | 0.55(0.34-0.90) | 0.016 |
|  |  |  | 70 |  | 0.03 | 0.04 | 0.64(0.36-1.11) | 0.111^#^ |
|  |  |  | 50 |  | 0.03 | 0.05 | 0.51(0.26-0.99) | 0.043 |
| 12 | IRS2 | rs1805097 | Total | C/T | 0.03 | 0.01 | 2.03(1.09-3.80) | 0.023 |
|  |  |  | 70 |  | 0.03 | 0.02 | 1.99(0.98-4.02) | 0.052^#^ |
|  |  |  | 50 |  | 0.03 | 0.02 | 1.43(0.63-3.26) | 0.386^#^ |
| 13 | LEPR | rs1805094 | Total | G/C | 0.09 | 0.12 | 0.74(0.56-0.99) | 0.039 |
|  |  |  | 70 |  | 0.10 | 0.12 | 0.80(0.57-1.12) | 0.191^#^ |
|  |  |  | 50 |  | 0.11 | 0.08 | 1.28(0.84-1.96) | 0.247^#^ |
| 14 | c9orf3 | rs3802457 | Total | G/A | 0.06 | 0.09 | 0.67(0.48-0.94) | 0.019 |
|  |  |  | 70 |  | 0.06 | 0.09 | 0.57(0.38-0.86) | 0.007 |
|  |  |  | 50 |  | 0.08 | 0.10 | 0.85(0.55-1.33) | 0.479^#^ |

| #- P value not significant |  |  |  |  |  |  |
| --- | --- | --- | --- | --- | --- | --- |

|  |  |  |  |  |  |  |  |  |
| --- | --- | --- | --- | --- | --- | --- | --- | --- |
|  |  |  |  |  |  |  |  |  |
|  |  |  |  |  |  |  |  |  |
|  |  |  |  |  |  |  |  |  |
|  |  |  |  |  |  |  |  |  |
|  |  |  |  |  |  |  |  |  |
|  |  |  |  |  |  |  |  |  |
|  |  |  |  |  |  |  |  |  |
|  |  |  |  |  |  |  |  |  |
|  |  |  |  |  |  |  |  |  |
|  |  |  |  |  |  |  |  |  |
|  |  |  |  |  |  |  |  |  |
|  |  |  |  |  |  |  |  |  |
|  |  |  |  |  |  |  |  |  |
|  |  |  |  |  |  |  |  |  |
|  |  |  |  |  |  |  |  |  |
|  |  |  |  |  |  |  |  |  |
|  |  |  |  |  |  |  |  |  |
|  |  |  |  |  |  |  |  |  |
|  |  |  |  |  |  |  |  |  |
|  |  |  |  |  |  |  |  |  |
|  |  |  |  |  |  |  |  |  |
|  |  |  |  |  |  |  |  |  |
|  |  |  |  |  |  |  |  |  |
|  |  |  |  |  |  |  |  |  |
|  |  |  |  |  |  |  |  |  |
|  |  |  |  |  |  |  |  |  |
