# Supplementary material for: The genetic susceptibility profile of type 2 diabetes and reflection of its possible role related to reproductive dysfunctions in the southern Indian population of Hyderabad
Source: BMC Med Genomics. 2021 Nov 16;14:272. doi: 10.1186/s12920-021-01129-0 (PMC8597259; doi:10.1186/s12920-021-01129-0)
Supplement: Supplementary file 5 — Additional file 5: Table S5. The results of logistic regression analyses of the 15 SNP earlier studied in the same cohort and the patterns of association with T2DM, unadjusted and adjusted for covariates [file 12920_2021_1129_MOESM5_ESM.docx]

**Additional file 5 Table S5: The results of logistic regression analyses of the 15 SNP earlier studied in the same cohort and the patterns of association with T2DM, unadjusted and adjusted for covariates**

| S.No | **Gene** | **SNP** | **MAF** | | **Major/Minor allele** | **CHISQ** | **Unadjusted** | | **Adjusted for covariates** | |
| --- | --- | --- | --- | --- | --- | --- | --- | --- | --- | --- |
|  |  |  | **Cases** | **Controls** |  |  | **OR** | **P value** | **OR** | **P value** |
| 1 | TCF7L2 | **rs7903146** | 0.34 | 0.21 | C/T | 41.89 | 1.97(1.61-2.43) | 9.65X10^-11^ | 1.95(1.52-2.50) | 1.58X10^-07^ |
|  |  | rs11196205 | 0.39 | 0.34 | G/C | 4.397 | 1.22(1.01-1.47) | 0.035 | 1.20(0.97-1.50) | 0.104^#^ |
|  |  | **rs12255372** | 0.24 | 0.18 | G/T | 10.94 | 1.45(1.16-1.81) | 0.001 | 1.42(1.09-1.83) | 0.008 |
| 2 | CDKAL1 | **rs7754840** | 0.27 | 0.2 | G/C | 10.49 | 1.42(1.14-1.75) | 0.001 | 1.37(1.06-1.77) | 0.015 |
|  |  | **rs7756992** | 0.29 | 0.22 | A/G | 11.19 | 1.42(1.15-1.74) | 0.001 | 1.41(1.10-1.81) | 0.007 |
| 3 | IRS1 | rs1801278 | 0.05 | 0.03 | G/A | 5.162 | 1.74(1.07-2.81) | 0.023 | 1.54(0.89-2.67) | 0.124^#^ |
| 4 | CAPN10 | rs3792267 | 0.16 | 0.13 | G/A | 4.158 | 1.30(1.01-1.68) | 0.041 | 1.55(1.14-2.10) | 0.004 |
|  |  | rs5030952 | 0.05 | 0.03 | C/T | 2.791 | 1.48(0.93-2.38) | 0.094^#^ | 1.1(0.62-1.92) | 0.739^#^ |
| 5 | CDKN2A/B | rs10811661 | 0.13 | 0.15 | T/C | 1.818 | 0.83(0.65-1.08) | 0.177^#^ | 0.82(0.61-1.15) | 0.210^#^ |
| 6 | HHEX | rs1111875 | 0.4 | 0.44 | A/G | 2.273 | 0.87(0.73-1.04) | 0.131^#^ | 0.93(0.75-1.15) | 0.549^#^ |
|  |  | rs7923837 | 0.44 | 0.45 | A/G | 0.393 | 0.94(0.78-1.13) | 0.530^#^ | 1.04(0.84-1.28) | 0.725^#^ |
| 7 | IGF2BP2 | rs1470579 | 0.5 | 0.49 | A/C | 0.481 | 1.06(0.89-1.27) | 0.488^#^ | 1.06(0.85-1.31) | 0.596^#^ |
|  |  | rs4402960 | 0.5 | 0.48 | G/T | 0.543 | 1.07(0.89-1.27) | 0.461^#^ | 1.06(0.85-1.31) | 0.620^#^ |
| 8 | PPARG | rs1801282 | 0.11 | 0.12 | C/G | 0.193 | 0.93(0.71-1.24) | 0.659^#^ | 0.92(0.67-1.28) | 0.651^#^ |
| 9 | SLC30A8 | rs13266634 | 0.22 | 0.22 | C/T | 0.342 | 1.07(0.86-1.32) | 0.558^#^ | 1.12(0.87-1.45) | 0.369^#^ |

**SNPs in bold indicate significant after multiple testing and # -p value not significant**
